# NAD substrate deficiency is an inherent and targetable risk factor for late-onset Alzheimer’s disease

**DOI:** 10.4103/NRR.NRR-D-25-00048

**Published:** 2025-05-06

**Authors:** Kai-Christian Sonntag, Bruce M. Cohen

**Affiliations:** McLean Hospital, Harvard Medical School, Boston, MA, USA

Sporadic or late-onset Alzheimer’s disease (LOAD) occurs in 1 of 10 people over 65 years of age and comprises 95% of all AD patients. Unlike early-onset AD, which is caused by defined single gene mutations, the mechanisms and events underlying risk for LOAD are not fully understood and no substantial disease-modifying interventions are currently available. Age is the most prominent risk factor for LOAD, and interacting age-related and LOAD-associated factors contribute to its pathogenesis. Among these factors are changes in bioenergetic cell functions, which metabolize substrates and produce energy stored in adenosine triphosphate. Our findings and the work of others have suggested that disturbances of these bioenergetic functions are both inherent and acquired during the aging process and contribute to LOAD dementia later in life (Ryu et al., 2021a, b; Cohen and Sonntag, 2024). Identifying these abnormal bioenergetic functions may lead to the development of agents or approaches to reduce the risk for LOAD.

**Figure 1:**
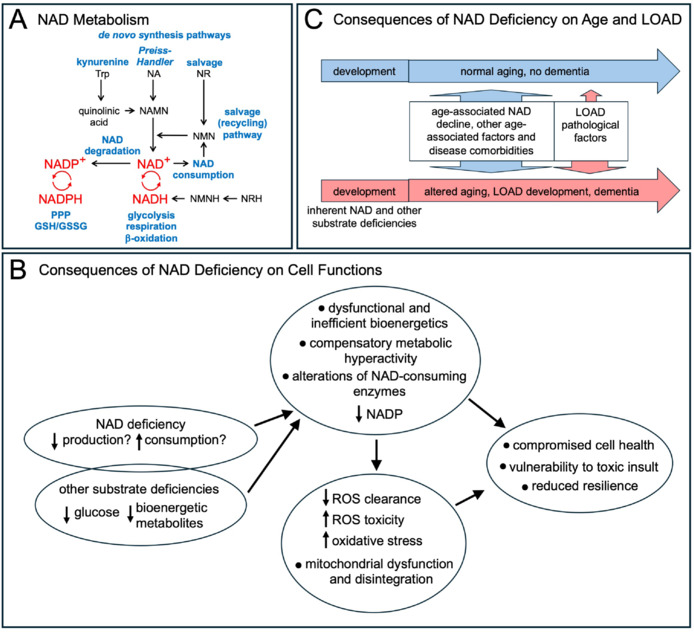
NAD metabolism and consequences of NAD deficiency. (A) Simplified schematic of NAD metabolism. NAD is *de novo* synthesized from tryptophan (Trp) in the kynurenine pathway producing quinolinic acid, from NA in the Preiss-Handler pathway, or from NR that enters the salvage pathway. In a recently discovered pathway, hydro-NR (NRH) is converted to NMNH which can be used to produce NADH. The pathways in the NAD metabolome are connected through the intermediate NAD precursors NAMN and NMN. NAD is degraded by NAD kinases to NADP and through NAD-consuming enzymes to nicotinamide, which enters the salvage pathway and is converted to NMN. While the NAD^+^/NADH redox cycle is an essential factor in glycolysis, mitochondrial respiration, and β-oxidation, NADP^+^/NADPH ratios are essential in the PPP and GSH/GSSG redox reactions. (B) Consequences of NAD deficiency with regards to cell functions. The reasons for age-related or inherent LOAD-associated NAD deficiencies are not well understood but could be a result of decreased NAD synthesis and/or increased NAD consumption. Concomitant to a reduction of NAD, deficits of other bioenergetic metabolites, such as glucose and lactate, are inherent to risk for LOAD and may occur with age. NAD together with other metabolic substrate deficiencies results in a reduction of NADP and dysfunctional and inefficient bioenergetic functions leading to compensatory metabolic hyperactivity and cell stress, diminished ROS clearance, increased ROS toxicity, oxidative stress, and mitochondrial dysfunction and disintegration. In addition, the activities of NAD-dependent enzymes are altered. These bioenergetic and metabolic changes compromise overall cell health, reduce cellular resilience, and increase vulnerability to toxic insults. (C) Consequences of NAD deficiency with regards to the aging process and the risk for development of LOAD. During age, a decline of NAD results in acquired compromises in cell health and vulnerability to other age-associated factors that impair health, including toxic cellular, environmental, and lifestyle factors. Disease comorbidities also increase the risk of unhealthy aging. However, the normal aging process does not need to lead to the development of dementia (blue). In contrast, LOAD-associated inherent NAD and other substrate deficiencies occur early in development and continue through youth and aging. They are additive with age-acquired NAD decline, thus, accelerating and worsening compromises in cell health and resilience during the aging process (red). These processes increase the vulnerability towards age-associated and LOAD pathological factors resulting in an altered aging process and the development of LOAD dementia. All individuals have some of the deficits associated with an increased risk for LOAD, but only those with the greatest number or effect of risk factors for LOAD develop dementia earlier in life, if they live into their 7^th^ decade. GSH: Glutathione; GSSG: glutathione disulfide; LOAD: late-onset Alzheimer’s disease; NA: nicotinic acid; NAD: nicotinamide adenine dinucleotide; NADP: nicotinamide adenine dinucleotide phosphate; NAMN: nicotinic acid mononucleotide; NMN: nicotinamide mononucleotide; NMNH: dihydronicotinamide mononucleotide; NR: nicotinamide riboside; NRH: dihydronicotinamide riboside; PPP: pentose phosphate pathway; ROS: reactive oxygen species; Trp: tryptophan.

An essential substrate in all bioenergetic processes is the redox cofactor nicotinamide adenine dinucleotide (NAD), which is in a constant state of synthesis, degradation, and recycling (Lautrup et al., 2024; **Figure 1A**). NAD levels are maintained either by *de novo* synthesis from tryptophan or via salvage pathways that utilize NAD precursors, such as nicotinamide, nicotinamide mononucleotide (NMN), nicotinic acid, and nicotinamide riboside (NR). In addition, NAD is converted to NADP by NAD kinases. Together, oxidized NAD^+^ and its reduced form, NADH, comprise the total NAD pool. NADH, which is mainly produced in glycolysis, the Krebs cycle, and β-oxidation, plays a critical role as an energy-transfer intermediate and is an important substrate of the mitochondrial electron transport chain, where it is oxidized to NAD^+^ by donating electrons and protons during oxidative phosphorylation. In normally functioning biosynthetic processes, the redox ratio (RR), measured as NAD^+^/NADH, is appropriately balanced and the electron transport chain is sufficiently powered for adenosine triphosphate production. In addition to serving as a redox agent, NAD is a co-enzyme consumed by numerous NAD-dependent factors that are critical to all cellular functions (Lautrup et al., 2024). Both NADH and NAD^+^ must be at adequate levels to serve these many reactions.

In the human brain, non-invasive *in vivo* measurements of NAD molecules using ^31^P or ^1^H magnetic resonance imaging methods have shown that during brain aging, total NAD and oxidized NAD^+^ gradually decrease, while NADH increases, leading to a reduction of the RR (Zhu et al., 2015; Bagga et al., 2020). These data are consistent with observations in non-human systems suggesting that a decline of NAD is a general and probably body-wide feature of aging organisms (Lautrup et al., 2024). The reasons why NAD declines with age are still incompletely understood but have been attributed to decreased levels or activity of NAD-synthesizing enzymes, such as nicotinamide mononucleotide transferase subtype 2 (NMNAT2) or nicotinamide phosphoribosyl transferase (NAMPT), or an increase of NAD-consuming enzymes (Lautrup et al., 2024).

In patients with LOAD, *in vivo* imaging and postmortem studies have also revealed bioenergetic abnormalities, and several groups, including ours, have studied bioenergetic mechanisms and factors in human subject-derived cell lines. To investigate metabolic cell functions relevant to LOAD, we used blood cells or skin fibroblasts from patients with LOAD and healthy control subjects to generate induced pluripotent stem cells (iPSC) that are then differentiated into immature cells, i.e., neural progenitor cells (NPCs), and mature brain cells, including neurons, astrocytes, oligodendrocytes, or microglia (Ryu et al., 2021a, b). This cell model can provide evidence on both inherent and acquired cellular risk factors associated with aging and LOAD, i.e., while the naïve skin cells can be used to investigate cellular factors in association with age, the iPSC paradigm models brain development. Since the iPSC reprogramming process eliminates epigenetic markers accumulated during life, abnormalities of cell functions in iPSC-derived cells are inherent, reflecting the effects of the many interacting genes underlying the risk for LOAD.

Our data have shown that iPSC-differentiated NPCs and astrocytes from patients with LOAD exhibit diminished total NAD levels and decreased RR (Ryu et al., 2021a, b). Reduced total NAD was also observed in LOAD patients-derived fibroblasts, but the RR was unchanged when compared to cells from young or old controls (Sonntag et al., 2017; Ryu et al., 2021a). Importantly, the NAD deficiencies are associated with a LOAD-specific bioenergetic cellular phenotype characterized by a general reduction of key bioenergetic substrates in combination with altered metabolic activity, as reflected by (1) decreased glucose uptake in response to insulin and insulin-like growth factor 1 signaling, (2) reduced insulin receptor and glucose transporter 1 densities, (3) diminished intracellular, but increased extracellular lactate levels, (4) elevated glycolytic rates with reduced capacity to process glycolytic substrates and lactate, and (5) heightened respiratory activity and oxygen consumption. Together, the data from both fibroblasts and iPSC-differentiated cells indicate that the identified bioenergetic alterations in LOAD are inherent cell-specific and cell-autonomous processes that may explain some mechanisms underlying and determining risk for LOAD (Cohen and Sonntag, 2024). Delineating the causal relationships among and between these abnormalities and NAD deficits remains an important goal of future studies.

Because a reduction of NAD has been associated both with age and age-related disorders, including neurodegenerative diseases, supplemental NAD has been suggested as an approach to improve overall cell health and for the treatment or prevention of LOAD. To this end, investigations in animal models of AD found that boosting NAD with its natural precursors (NR or NMN) or with P7C3, an allosteric activator of NAMPT, improved cellular processes associated with AD pathology, including reduced amyloid and Tau pathologies; improved mitochondrial function, mitophagy, DNA repair, and autophagy; increased stem cell regenerative activity in the hippocampus; and reduced signs of inflammation and senescence (Lautrup et al., 2024). Based on these findings, clinical trials with NAD supplementation have been conducted or are currently underway in patients with mild cognitive impairment or AD. Although there has been some evidence that boosting NAD can improve cognitive function and alter AD pathological markers, the available data are too limited to conclude that this approach is an effective pharmacological intervention for LOAD (Alghamdi and Braidy, 2024; Lautrup et al., 2024).

We found that treatment of iPSC-derived NPCs and astrocytes with NR in combination with caffeine, which augments NMNAT2 activity, partially restored NAD levels in LOAD cells and enhanced some aspects of bioenergetic cell functions. However, it did not markedly alter the LOAD-associated bioenergetic phenotype described above (Ryu et al., 2022). Importantly, while NR and caffeine could normalize NAD levels in LOAD NPCs to levels like those seen in control NPCs, this was not the case in LOAD astrocytes, which still had about 40% lower NAD and no substantial improvement of dysfunctional bioenergetic functions with treatment.

We propose that NAD deficiency and associated bioenergetic dysfunctions are inherent cell-specific and cell-autonomous features underlying LOAD pathology. NAD deficiency is seen across cell types, while the response to supplements is cell-type specific. That finding is important in designing future human studies targeting NAD levels. Diminished levels of NAD in LOAD iPSC-derived immature and mature brain cells seemingly go together with other bioenergetic dysfunctions, including reduced uptake and availability of glucose and other key bioenergetic substrates along with a compensatory metabolic hyperactivity. Altogether, LOAD cells are compromised in bioenergetic activity and the ability to produce more energy. Their metabolic hyperactivity, even without stress, implies that they are working harder at baseline to maintain energy homeostasis.

The consequences of these inter-related metabolic dysfunctions are diverse (**Figure 1B**). Low levels of NAD not only impair redox reactions but also impact cellular capacity to respond to oxidative stress (Jomova et al., 2023). At the same time, intensive energy metabolism leads to the production of reactive oxygen species (ROS), which have toxic effects that damage mitochondria, proteins, lipids, and DNA. Low NAD also causes a reduction of its derivative NADP/H, which is required to convert the oxidized free radical scavenger glutathione, GSSG, back to its reduced form, GSH. GSH, in turn, is needed to reduce ROS. Altogether, reduced NAD, NADP, and GSH predispose to mitochondrial dysfunction and damage. Damaged mitochondria often induce cell death, through compromised cell functions, in general, and apoptosis, in particular (Jomova et al., 2023).

Other substrates in the NAD metabolome have also been implicated as being dysregulated in LOAD. For example, metabolites in the kynurenine pathway have been linked to neurodegeneration, through neurotoxicity and increased oxidative stress (Pathak et al., 2024). In addition, low NAD compromises a series of NAD-dependent enzymes that catalyze the formation of nicotinamide and adenosine diphosphate ribose (ADPR) or cyclic ADP-ribose (cADPR) from NAD^+^. These enzymes and their bioenergetic-related byproducts are involved in many of the cellular functions that play a part in the general aging process and LOAD pathology (Lautrup et al., 2024). Examples relevant to brain cells in which adequate NAD levels are crucial include: (1) poly (ADP-ribose) polymerase activity, which has a key role in DNA repair, genomic stability, and apoptosis; (2) activity of members of the sirtuin gene family, which are involved in a wide range of cellular processes, such as gene transcription, sensing caloric fluctuations, autophagy, and stress resistance; (3) CD38 activity that metabolizes NAD and NADP and participates in calcium signaling; and (4) sterile alpha and TIR motif containing 1 activity, a key regulator in axon degeneration.

Finally, our findings of NAD deficiency and associated bioenergetic and metabolic dysfunction in iPSC-derived astrocytes have profound implications for the metabolic activity of the brain at large. Astrocytes consume about 80 percent of the blood-derived glucose of the brain, which is converted to lactate through glycolysis and used via the astrocyte-neuron lactate shuttle as a fuel source in neurons. Neurons in turn are highly dependent on oxidative phosphorylation and the metabolism of lactate to drive respiration. In addition, energy-dependent astrocyte activity is essential in recycling glutamate that would otherwise be toxic at glutamatergic synapses, the most common synapses in the brain. Thus, bioenergetically inefficient or stressed astrocytes may play a central role in dysfunctional brain metabolism and cell death associated with LOAD pathology. And, as suggested by our data, attempts to boost NAD may not be successful in these cells, which could be one of the reasons why NAD supplementation has not produced substantial improvement in cognitive brain functions in patients with LOAD. There may be other ways to target and support NAD levels and use them in astrocytes and other brain cells.

LOAD is a complex disorder characterized by multiple interacting inherent and environmental factors. In addition to LOAD-associated inherent genetic variants and cell functional risk factors, acquired factors include aging and disease comorbidities, such as trauma, cardiovascular disease, and type 2 diabetes mellitus, as well as lifestyle factors, especially diet, exercise, and psychosocial stimulation, all of which can alter energy metabolism (Cohen and Sonntag, 2023, 2024). NAD substrate deficiency and associated bioenergetic dysfunctions are inherent risk factors of LOAD, which are amplified by age-acquired NAD reductions, changes in NAD redox, and compromised energy metabolism (**Figure 1C**). While young, these bioenergetic deficiencies can be compensated for, but in middle to old age, the amount of NAD substrate may become quantitatively insufficient to maintain metabolic needs or provide sufficient compensatory reserve in situations of cell stress. Thus, with inherently low and age-related further reductions of NAD, there is a gradual acceleration of cellular bioenergetic decompensation that eventually leads to disintegration and cell death, and untimely onset of dementia. Although NAD has been suggested as a targetable factor in LOAD, our results and other data indicate that simple supplementation of NAD may not affect every cell type equally or adequately and may not address various NAD- and LOAD-associated dysfunctional bioenergetic and other metabolic pathways. To establish if increasing NAD levels and effects are feasible targets for treating, preventing, or delaying the onset of LOAD, more work is necessary to identify the cause and consequences of NAD deficiency associated with LOAD risk and pathology. The technologies to answer these questions are available (Ryu et al., 2021a). We suggest that such studies are timely and promising.
